# SCOPE: predicting future diagnoses in office visits using electronic health records

**DOI:** 10.1038/s41598-023-38257-9

**Published:** 2023-07-07

**Authors:** Pritam Mukherjee, Marie Humbert-Droz, Jonathan H. Chen, Olivier Gevaert

**Affiliations:** 1 Department of Medicine, Stanford Center for Biomedical Informatics, Stanford University, 1265 Welch Rd, Palo Alto, CA 94305 USA; 2 Department of Biomedical Data Science, Stanford University, Palo Alto, CA USA

**Keywords:** Machine learning, Outcomes research, Predictive markers, Statistics

## Abstract

We propose an interpretable and scalable model to predict likely diagnoses at an encounter based on past diagnoses and lab results. This model is intended to aid physicians in their interaction with the electronic health records (EHR). To accomplish this, we retrospectively collected and de-identified EHR data of 2,701,522 patients at Stanford Healthcare over a time period from January 2008 to December 2016. A population-based sample of patients comprising 524,198 individuals (44% M, 56% F) with multiple encounters with at least one frequently occurring diagnosis codes were chosen. A calibrated model was developed to predict ICD-10 diagnosis codes at an encounter based on the past diagnoses and lab results, using a binary relevance based multi-label modeling strategy. Logistic regression and random forests were tested as the base classifier, and several time windows were tested for aggregating the past diagnoses and labs. This modeling approach was compared to a recurrent neural network based deep learning method. The best model used random forest as the base classifier and integrated demographic features, diagnosis codes, and lab results. The best model was calibrated and its performance was comparable or better than existing methods in terms of various metrics, including a median AUROC of 0.904 (IQR [0.838, 0.954]) over 583 diseases. When predicting the first occurrence of a disease label for a patient, the median AUROC with the best model was 0.796 (IQR [0.737, 0.868]). Our modeling approach performed comparably as the tested deep learning method, outperforming it in terms of AUROC (p < 0.001) but underperforming in terms of AUPRC (p < 0.001). Interpreting the model showed that the model uses meaningful features and highlights many interesting associations among diagnoses and lab results. We conclude that the multi-label model performs comparably with RNN based deep learning model while offering simplicity and potentially superior interpretability. While the model was trained and validated on data obtained from a single institution, its simplicity, interpretability and performance makes it a promising candidate for deployment.

## Introduction

Widespread adoption of electronic health records (EHR) has offered great potential for learning and applying from real-world data streams, while simultaneously burdening practitioners with documentation clerical work that takes away from direct patient care. Primary care physicians may spend as much as one-half of their workday interacting with the EHR^1^, reducing the time dedicated to patient care^2^. Further, the documentation load may reduce the satisfaction of physicians and may even lead to burnout^3^. Additionally, EHR data is often biased^4^ and suffers from missing and incomplete data^5,6^. Here we seek to develop machine learning methods to address these key challenges to unlock the potential of EHRs in the outpatient office visit setting.

The key focus of our work is predicting likely diagnoses for patients from past medical history. In recent years, there has been a body of work on the prediction of diagnoses and patient outcomes from past medical history obtained from EHRs^7–9^. Our work focuses exclusively on outpatient visits. In terms of methods, recent work has focused heavily on deep learning approaches^10^; here however, we propose classical machine learning models such as logistic regression and random forests which offer much greater interpretability as well as modularity and scalability. Finally, instead of focusing on only one or a few diseases^11^, we evaluate these models across a wide range of diseases at an appropriate level of granularity, subject to data constraints. This is motivated by the fact that patients in the outpatient clinic often present with multiple chronic and acute diseases, and while single disease models are very useful, it quickly becomes cumbersome to maintain and get meaningful predictions from multiple disparate models. We present a unified approach to modeling the broad scope of practice in the outpatient clinic.

An important consideration for machine learning analysis of EHRs is interpretability. By interpretability^12^, we mean not only post hoc explainability of predictions, but also algorithmic transparency and model decomposability whereby each step of the inference process including the inputs, the parameters and the computations can be interpreted by humans. This is in contrast to deep learning models which may be post hoc explainable, e.g. using Shapley additive explanations^13^, but are still “black boxes" in terms of the parameters and computational steps during inference, and may not be suitable for clinical use^14^. Interpretable models such as linear models and decision trees behave predictably, are typically more robust and allow deep inspection when biases or discrepancies are observed, thus engendering trust among physicians.

We propose to use machine learning to lessen the physician burden by developing a machine learning model that can predict the likely cause of an office visit using missing or incomplete EHRs data from outpatient office visits. For this approach, we do not confine ourselves to a single disease specialty but rather aim at developing a broadly applicable tool. To that end we developed SCOPE (SCalable One-vs-all PrEdictor) an inference engine powered by an interpretable machine learning model, that predicts likely diagnoses for an office visit encounter based on the patient’s past medical history. Before the visit, defined as the pre-visit, SCOPE predicts the likely diagnoses for a patient based on the data of their previous visits and explains its predictions by highlighting the features responsible for its predictions. This can help the physicians plan out the encounter more efficiently, potentially saving them time and effort. In the post-visit documentation stage, the model can act like a recommender system, helping physicians fill out the diagnoses for the visit. This can reduce the likelihood of missing or incomplete diagnoses records. Using a binary relevance based multi-label modeling approach, we developed a model that is extensible, interpretable, and shows state-of-the-art predictive performance on a large EHR dataset obtained from Stanford hospital and clinics. We show that these models perform at par or better than the popular deep learning methods^15–18^.

## Materials and methods

### EHR data processing

**Figure 1:**
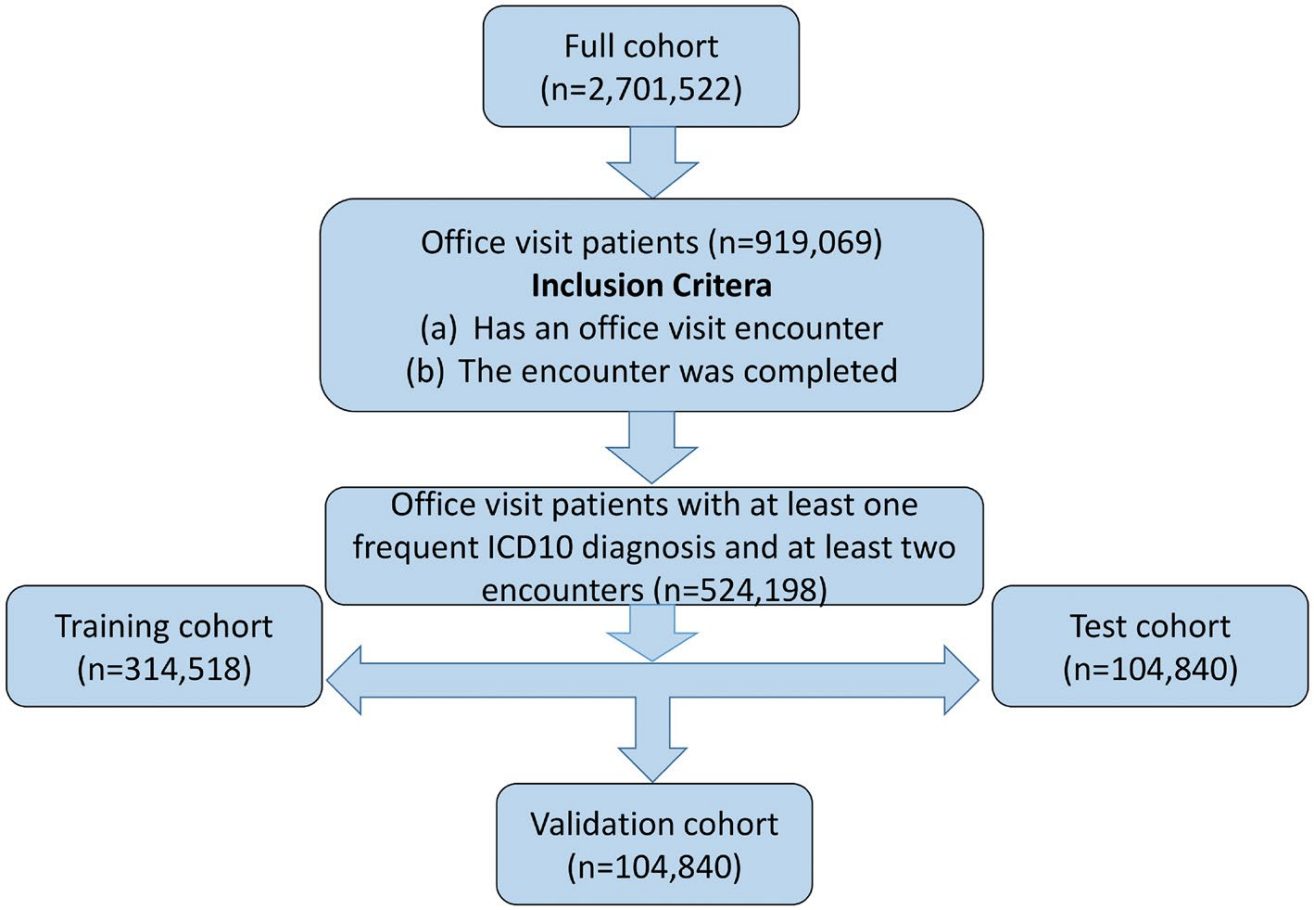
Cohort selection for this study.

This study was approved by the Stanford IRB under protocol IRB-50033 “Machine Learning of Electronic Medical Records for Precision Medicine”. Consent for this study was waived by the IRB. For this study, the EHR data of 2,701,522 patients at Stanford Healthcare with 55,068,909 encounters over a time period from January 2008 to December 2016 was retrospectively collected and deidentified in accordance with approved IRB guidelines. We selected all encounters that were completed office visits with a “frequent" (present in at least 500 patients) ICD-10 diagnosis code (see Fig. 1). The full set of patients were split into a training (60%), a validation (20%) and a testing (20%) set. All models are trained on the training set; the performance on the validation set is used for model selection or choosing modeling parameters such as the aggregation window. The testing set is used only for the final performance evaluation.

### Input features and output labels

The output labels at each encounter were the corresponding ICD-10 diagnostic codes. The ICD-10 codes start with a letter (letter prefix) which roughly categorize the disease type or affected system (Supplementary Table 1); for e.g. codes starting with I correspond to diseases of the circulatory system. We excluded codes starting with R, U, V, W, X, Y and Z, since they comprise either symptoms, codes for special purposes, external causes of morbidity and mortality or factors influencing health status and contact with health services. To avoid the large cardinality of all possible ICD-10 diagnosis codes, we grouped codes under their 3-character prefixes, for example, all codes starting with I25 are assigned the same label I25. Each label is further designated as acute or chronic using the Chronic Condition Indicator for ICD-10-CM^19^.

In order to predict the probable diagnoses at an encounter, we considered patient demographics (age and sex), past diagnoses codes, and lab results. We considered four time windows for diagnosis codes: 90, 180, 365 and 3650 days, and four time windows for lab results: 30, 90, 180 and 365 days. We built models based on both diagnostic codes and lab results individually for each of the respective aggregation windows. The best time windows were chosen for both diagnostic codes and lab results and were used for multi-modal feature integration in the next step.

### Modeling strategy

We adopted a binary relevance based multi-label classification strategy using either logistic regression (LR) or random forest (RF) as the base classifier. In this framework, we train $L$ different logistic regression or random forest models ${M}_{l}$, one for each label $l, l=1,\dots , L$, based on a preprocessed dataset ${X}_{l}$, obtained from the same feature set $X$. During inference, the input features are preprocessed appropriately for each label and the corresponding model predicts the presence or absence of the label given the input. In our case, for the LR models, the preprocessing for training includes a majority under-sampling step (1:1 positive to negative ratio), followed by a maximum absolute scaler, followed by a logistic regression classifier with an ${l}_{1}$ or ${l}_{2}$ penalty. The processing pipeline for the RF models consists of the majority under-sampling step, followed by a random forest classifier.

After selecting the best time windows for both diagnostic codes and lab features, we used an early stage integration strategy where the respective feature vectors are concatenated to form a longer vector in order to combine features from multiple modalities (i.e. demographics, diagnosis codes and lab results). The best model is selected among the different combinations of estimators (LR or RF) and input modalities. To evaluate and compare the efficacy of different models, we primarily use the Area under the Receiver Operating Characteristics Curve (AUROC) since it is insensitive to varying class imbalance across different labels. For the final model, we also employ other metrics including the Area under the Precision Recall Curve (AUPRC), recall$@$k (see Supplementary Information for details) and coverage error, and evaluate its performance for de novo predictions, i.e., the performance at predicting the first occurrence of the a given label. For this we only consider encounters till the first occurrence of a given label, for each patient—thus, subsequent occurrences of the label (which may be easier to predict) are not considered when evaluating the performance of the model in this case. Finally, the model was calibrated using isotonic regression^20^ on the training set, and interpreted using the SHAP (SHapley Additive exPlanations) framework^13^.

### Comparison with deep learning

We implemented the deep learning architecture proposed in Choi et al.^15^. It was designed for the same problem of predicting future diagnoses for a broad range of diseases in the outpatient setting, and thus, we can directly compare the performance of our modeling approach to it. To ensure a fair comparison, we trained the deep learning model (DL) to predict future diagnoses using past diagnosis codes only and compared it to multi-label classifiers using LR and RF as the base classifier trained on diagnosis codes only. We adopted the best performing model architecture in Choi et al.^15^ as the reference: an RNN with two hidden layers, and experimented with various embedding dimensions (i.e. 100, 500, 1000, 1500 and 2000), the number of past encounters considered in the input (10, or 20), and initialization of the embedding layer with random vs Skip-gram^21^ embeddings. We compared the performance of the best performing DL model with the proposed LR and RF based pipelines.

### Modeling, analysis and visualization

Most of the modeling tasks performed in this paper, including cleaning the data, developing the LR and RF models, as well as model calibration and interpretation were performed in Python 3.6 using various packages including numpy^22^, pandas^23^, scikit-learn^24^, matplotlib^25^, imbalanced-learn^26^, pint, and WorldCloud. The statistical analysis and plotting was performed in R. The Wilcoxon signed rank test^27^ is used for comparing between models, unless otherwise stated. In particular, the ggpubr package was used for several of the plots.

## Results

### Stanford cohort

Out of 2,701,522 patients in the original data, 919,069 patients had a completed office visit encounter. Removing encounters and patients with no ICD-10 diagnostic codes reduced the number of patients to 752,734. At this stage, there were 24,617 unique ICD-10 codes. Dropping infrequent codes (occurring for fewer than 500 patients) and patients with only one encounter reduced the number of patients to 524,198 and the number of ICD-10 codes to 2045 (see Table 1 for summary statistics of the selected cohort).

**Table 1:** Summary statistics of the Stanford cohort.

	Total	Train	Validation	Test
Number of patients	524,198	314,518	104,840	104,840
Male	232,789 (44%)	139,660 (44%)	46,489 (44%)	46,640 (44%)
Female	291,377 (56%)	174,839 (56%)	58,344 (56%)	58,194 (56%)
Unknown	32 (0%)	19 (0%)	7 (0%)	6 (0%)
Number of encounters	5,332,739	3,196,459	1,064,381	1,071,899
Number of encounters per patient				
Median	5	5	5	5
5% quantile	2	2	2	2
95% quantile	34	34	34	34

The original labs data for the chosen 524,198 patients had 13,891 unique lab names. After harmonizing the lab names and dropping the infrequent labs (occurring in fewer than 500 patients), we ended up with 1504 labs comprising 356 nonnumeric and 1148 numeric labs. The values for each of these 1504 labs were harmonized to have consistent units for numeric labs and binary values for the nonnumeric labs; overall less than 5% of the lab result instances were dropped during this harmonization process. In terms of output labels, out of 1460 relevant three-character ICD-10 codes (after dropping R and U to Z codes), the final cohort covers 583 labels.

### Aggregating past encounters improves predictive performance

For diagnosis codes, we tested four time windows for aggregation: 90, 180, 365 and 3650 days. For lab results, the four time windows were 30, 90, 180 and 365 days (Supplementary Fig. 1). For both cases, increasing the length of the aggregation windows significantly improved the overall AUROC performance (p $<0.001$) on the validation set. Thus, we chose the longest aggregation windows: 3650 days for diagnostic codes and 365 days for the lab results for further model development.

### Integration of diagnostic codes, lab results and demographic data

Models were developed using diagnostic codes alone (Diag), lab results alone (Labs), a combination of lab results and diagnostic codes (LabsDiag) and the combination of lab results, diagnostic codes and demographic features (LabsDiagDemo) (Supplementary Fig. 2). The LabsDiagDemo models perform best in terms of average AUROC. For both LR and RF, using diagnostic codes only leads to significantly better performance than using lab results only (median AUROC 0.896 vs 0.758, p $<0.001$). For LR, combining the diagnostic codes and lab results slightly improves the AUROC performance further from 0.893 to 0.894 (Supplementary Fig. 2). For RF, however, the performance with LabsDiag is statistically comparable (at p $=0.05$) as that of Diag. Adding the demographic features slightly but significantly improves the performance (0.899 vs 0.894, and 0.906 vs 0.904, Supplementary Fig. 2) on the validation set for both LR and RF models. Thus, we use the combination of diagnostic codes, lab results and demographic features (LabsDiagDemo) for further model development and analysis.

### Random forests outperforms logistic regression

**Figure 2:**
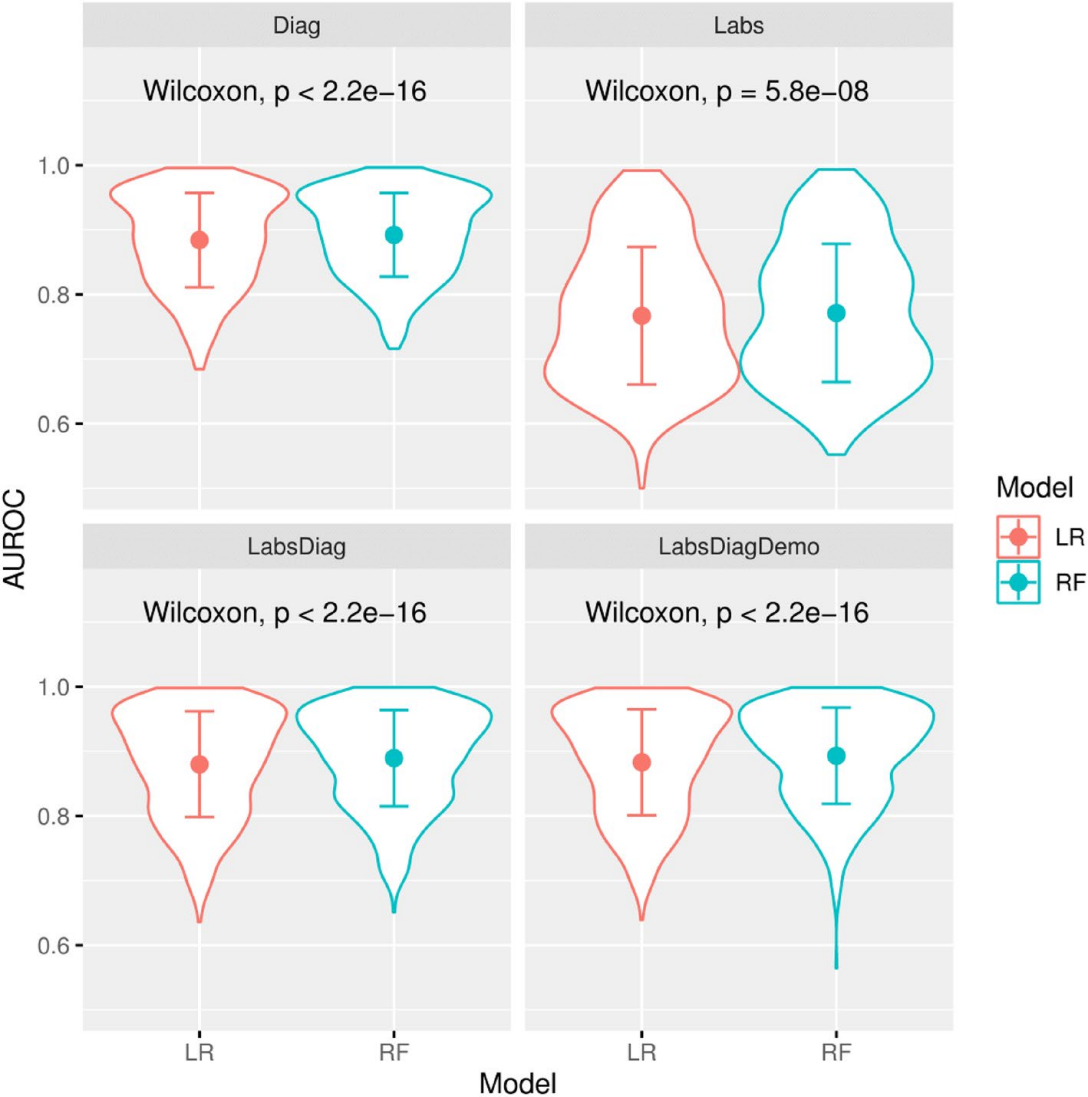
Violin plots showing the AUROC performance comparison between logistic regression (LR) and random forest (RF) with different inputs: diagnostic codes only (Diag), lab results only (Labs) integration of both diagnostic codes and lab results (LabsDiag) and integration of diagnostic codes, lab results and demographic features (LabsDiagDemo). The p-values obtained by a paired Wilcoxon signed-rank test (top of the plots) shows that RF outperforms LR significantly in every case.

The LR and RF models developed on Diag, Labs, LabsDiag and LabsDiagDemo were compared in terms of AUROC performance (Fig. 2). On LabsDiagDemo, RF significantly outperforms LR, both overall (median AUROC 0.906 vs 0.899, p $<0.001$), and for most individual letter prefixes as well (Table 2). Thus, RF is chosen as the best approach for subsequent model development and analysis.

**Table 2:** Breakdown of average AUROC for LR and RF models trained on LabsDiagDemo by ICD-10 letter prefixes. The letter prefixes represent groupings of diagnostic codes (roughly organized by disease type and affected systems, for e.g., codes prefixed with I represent diseases of the circulatory system) in the ICD-10 coding system. The p-value is obtained using Wilcoxon signed rank test with alternative hypothesis RF outperforming LR, and then are adjusted for multiple hypothesis tests using the Benjamini–Hochberg procedure with false discovery rate < 0.05. The highlighted cells indicate that the p < 0.05.

Letter prefix	Label_count	LR	RF	p value
A	10	0.832	0.848	3.36E−02
B	21	0.844	0.858	7.60E−03
C	28	0.977	0.972	1.00E+00
D	35	0.889	0.903	7.98E−05
E	34	0.915	0.927	4.04E−05
F	37	0.929	0.934	6.11E−02
G	30	0.908	0.918	1.93E−03
H	42	0.853	0.872	2.98E−05
I	44	0.919	0.926	2.09E−03
J	31	0.861	0.871	1.94E−03
K	45	0.849	0.864	2.98E−05
L	44	0.821	0.842	9.50E−06
M	53	0.886	0.897	9.50E−05
N	51	0.898	0.907	1.67E−04
O	7	0.981	0.979	7.82E−01
P	3	0.995	0.994	7.82E−01
Q	16	0.939	0.935	1.00E−00
S	38	0.821	0.832	2.94E−03
T	14	0.809	0.810	4.31E−01

Next, the RF pipeline is used to develop calibrated models using isotonic regression with five-fold cross-validation on the training set. The calibrated model was used to predict on the validation and test sets. The model shows good calibration for most labels (Supplementary Fig. 3).

### Interpretation of the model

The RF based multi-label model is essentially a collection of independent RF models, one for each disease label; therefore to interpret predictions for a particular label, it suffices to consider only the corresponding model. Each RF model is an ensemble of decision trees which are algorithmically transparent, i.e., during inference, the prediction path, consisting of several binary comparisons, can be traced from the inputs (which are trivially interpretable without any preprocessing or embedding). This is quite unlike deep learning models in which the inference path cannot be meaningfully interpreted or traced.

Chapter level feature importances for a select few cases were visualized using word clouds (Supplementary Fig. 4). For several letter prefixes such as C: neoplasms, F: mental and behavioural disorders, I: diseases of the circulatory system, and J: diseases of the respiratory system, we can observe that diagnostic codes figure prominently as important features for every letter prefix; in particular, codes belonging to the same letter prefix.

We also visualized important features (based on the SHAP framework, see Supplementary Information for details) for individual labels (Supplementary Fig. 5). For example for lung cancer, the important features include prior history of lung cancer, other abnormalities in the lung, age, presence of glucose measurements as well as blood counts. Blood count tests are routinely ordered for lung cancer patients to monitor overall health and aid in treatment decisions.

Next, for bipolar disorder, a history of bipolar disorder and age appear as important features. In addition, we see codes for several related disorders such as major depressive disorder, anxiety disorder, hypertension, insomnia which are known to be associated with bipolar disorder^28,29^. Notably, we also see tests for lithium, tricyclics and valproic acid which are used to treat bipolar and depressive disorders.

For heart failure, besides a history of heart failure, we prominently see age and natriuretic peptide tests (BNP and NT-proBNP) which are used to diagnose heart failure. We can also observe several factors that are known to be associated with higher incidence of heart failure: presence of atrial fibrillation^30^, atherosclerotic heart disease and higher QRSD interval^31^, higher heart rate^32^, higher red cell distribution width^33^, lower estimated globular filtration rate (eGFR)^34^ and hypertension which is often a precursor of heart failure^35^.

Finally, for chronic obstructive pulmonary disease (COPD), codes indicating a history of COPD, nicotine dependence, asthma or dyspnea appear as important features. Additionally, we observe the well-known association of COPD with chronic kidney disease^36^ through kidney function lab tests such as eGFR, blood urea nitrogen (BUN), and creatinine, and heart diseases such as atherosclerosis and heart failure^37^. Overall, the model selects meaningful and interpretable features for predicting these diagnoses.

### Comparing with the deep learning approach

We compared the performance of our modeling approach to the deep learning proposed in Choi et al.^15^ In terms of overall AUROC performance across all labels, both the LR and RF multi-label classifiers trained on diagnostic codes only outperform the best deep learning model (p $<0.001, \mathrm{data not shown}$). In terms of AUPRC however, the DL model performs significantly better across all letter prefixes (p $<0.001, \mathrm{data not shown}$).

**Figure 3:**
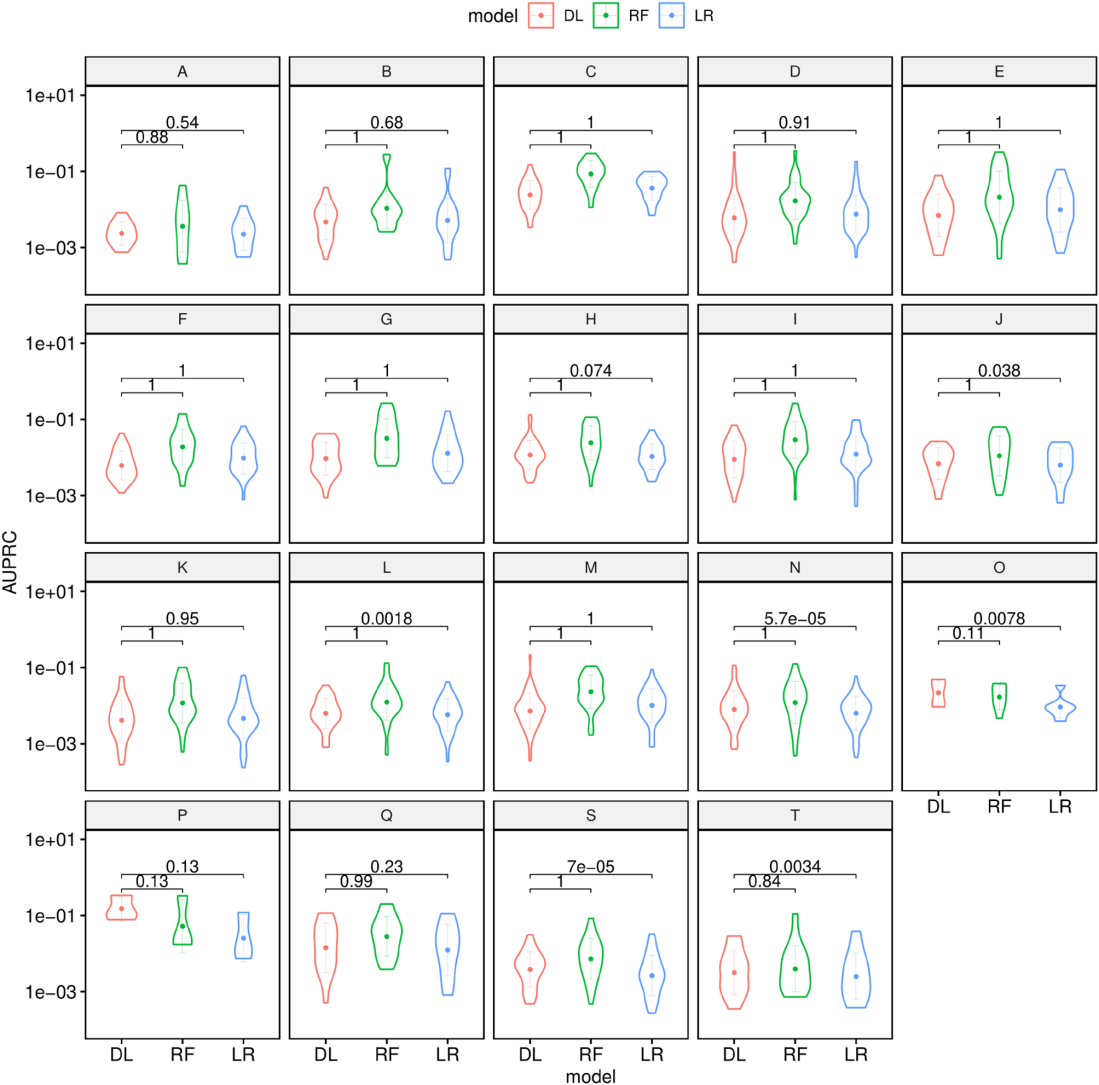
Violin plots showing the chapterwise AUPRC Performance of DL vs LR vs RF on diagnostic codes for de novo predictions. The numbers atop the plots indicate the p-values obtained by a paired Wilcoxon signed-rank test with the alternative hypothesis that DL is better on overage.

The situation reverses for de novo predictions: the overall AUROC performance of the DL model is better across all letter prefixes (p $<0.001$, Fig. 3, Supplementary Fig. 6) than the LR and RF based models; however, the AUPRC performance is significantly worse (p $<0.001$).

### Model calibration and comparison with existing literature

**Figure 4:**
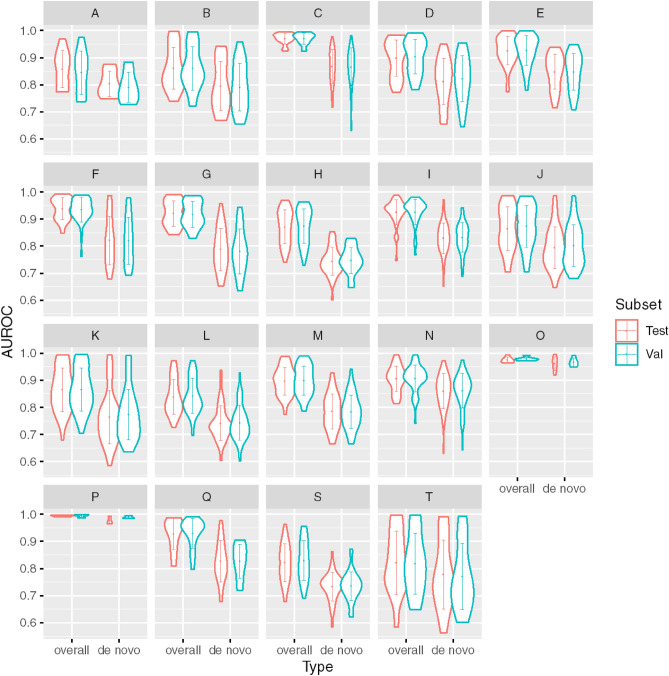
Violin plots showing the chapter-wise AUROC performance of the calibrated model on the validation and test sets for all encounters (overall) and de novo encounters.

**Figure 5:**
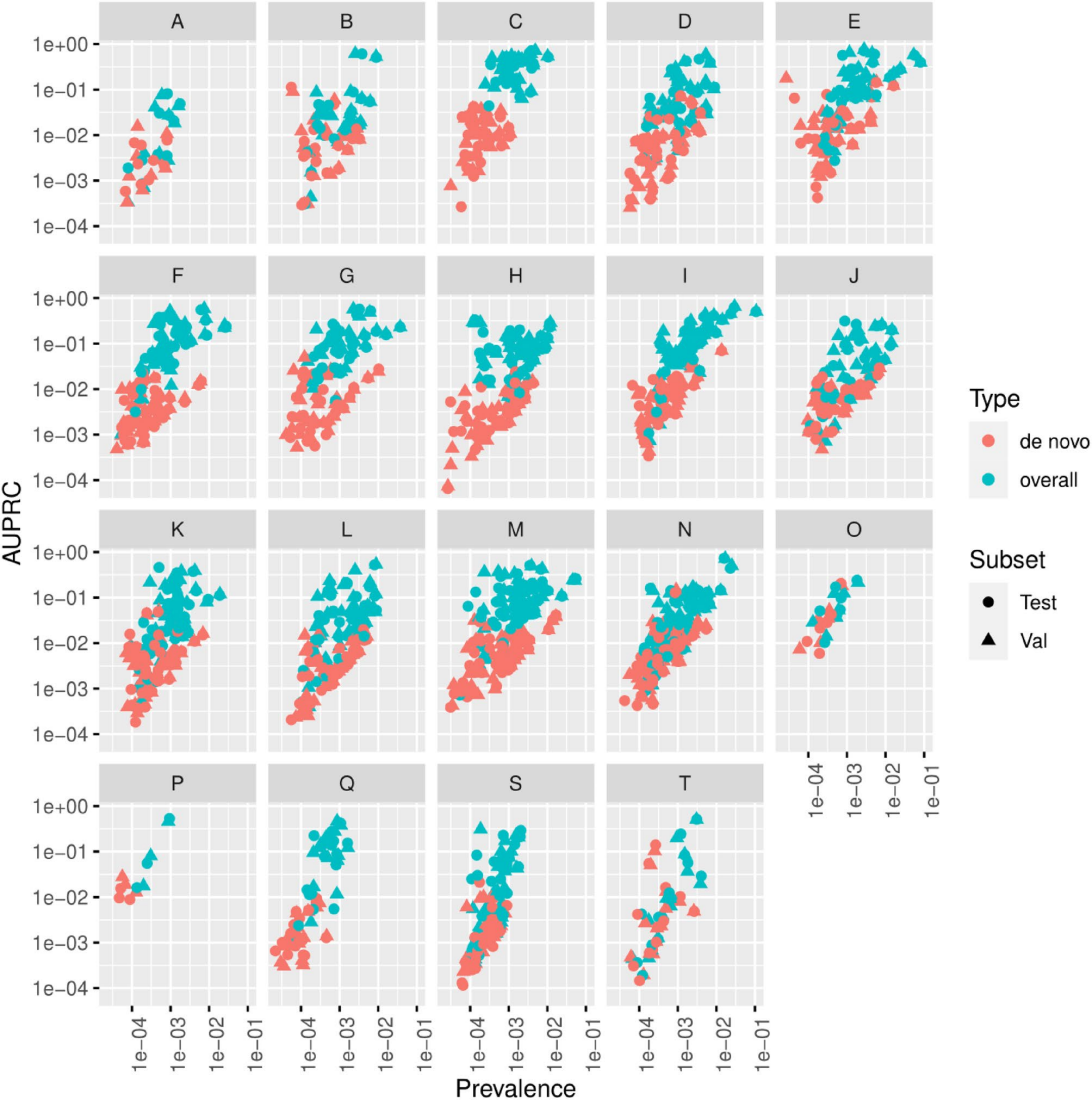
Scatter plots showing the chapter-wise AUPRC performance of the calibrated model on the validation and test sets as a function of prevalence for all encounters (overall) and de novo encounters.

Next, the performance of the calibrated model was assessed on the validation and test sets in terms of AUROC (Fig. 4) and AUPRC (Fig. 5) and comparing overall and de novo predictions. We observe that the predictive performance of the model is better on overall predictions vs. de novo encounters (Figs. 4, 5). This difference in AUPRC can be attributed to increased class imbalance; when only de novo encounters are considered for a label, the prevalence decreases, and the AUPRC decreases correspondingly. The model also performs significantly better (median overall AUROC 0.934 vs 0.848, p $<0.001$) on chronic disease vs. acute diseases. The overall AUROC performance on chronic diseases such as neoplasms and mental and behavioral disorders is superior to the performance on letter prefixes with more acute diseases such certain infectious and parasitic diseases injury, poisoning and certain other consequences of external causes.

Next, we compared the RF and DL models with two previous studies using DL: Choi et al.^15^ and Rashidian et al.^11^ In terms of the recall$@k$ metric, the RF model performs similarly with the results reported in those reported by Choi et al. on their data. However, their DL model trained with diagnostic codes on Stanford data performs worse (Table 3). Secondly, we compared our results for three diseases studied by Rashidian et al.—in each case, the AUROC performance of the RF model exceeds the results reported in Rashidian et al., as well as the DL model trained on Stanford data.

**Table 3:** Performance of the final model, and comparison with existing literature. The DL model was trained on diagnostic codes only.

Performance metric	RF model		DL model		Previous results^19,23^
	Validation	Test	Validation	Test	
Comparison with Choi et al.^23^					
Recall					
@10	0.646	0.643	0.504	0.503	0.643
@20	0.748	0.746	0.612	0.612	0.743
@30	0.800	0.799	0.675	0.674	0.796
Comparison with Rashidian et al.^19^					
AUROC					
Acute renal failure (N17)	0.936	0.922	0.900	0.889	0.919
Chronic kidney disease (N18)	0.973	0.970	0.940	0.939	0.942
Diabetes mellitus (E10, E11, E13)	0.976	0.972	0.922	0.917	0.915

## Discussion

In this paper, we have developed SCOPE a model for predicting ICD-10 diagnoses codes for a patient at a given encounter, based on the patient’s demographics, past diagnoses and lab test results. We adopted a binary relevance based multi-label modeling approach that is easily extensible and allows us to inspect and interpret the models for each individual label separately. SCOPE showed good calibration in the held-out test set and performed comparably or better with other models in the literature. The final model, SCOPE was a binary relevance based classifier with a RF trained on the concatenation of aggregated diagnostic codes, lab results and demographic features.

The proposed model predicts three-character prefixes of ICD-10 codes instead of the full code. In practical use, we envision a human-in-the-loop setup where the model suggests the three-character code prefix and the physician adds the relevant suffix. There are several advantages of this choice. First, for many codes, it is not possible to specify the exact code accurately just based on past medical history—for example, the codes under F31 (bipolar disorder) specify the severity of the disease at the current encounter and requires evaluation by the physician based on other factors such as current symptoms. Second, the exact ICD-10 billing code used in a situation can vary based on the physician’s subjective opinion and the medical institution’s conventions—the three-character prefix is more robust to such variation and the model will likely generalize better. Finally, from a technical standpoint, it is beneficial to reduce the cardinality of the target labels and achieve better performance.

Our work is similar in spirit to several existing papers in the literature. With the widespread adoption of EHR and the availability of large datasets, deep learning approaches have been used for most EHR based modeling and prediction tasks such as learning patient or context representation^16,18^, outcome prediction^7,8,38–40^, and prediction of future diagnoses^11,17,41^ in recent years^10,42,43^. In particular, Choi et al.^15^ used a recurrent neural network (RNN) model to predict diagnosis codes from past diagnosis codes and medication data obtained from the EHR, and showed it outperformed a baseline logistic regression model. Indeed in our initial experiments, we found that a naïve logistic regression does not perform well on the prediction task. In contrast, we find that a binary relevance based multi-label strategy with logistic regression as the base model outperforms the RNN model of Choi et al.^15^ We hypothesize that this disparity is caused by the two factors: first, the high class imbalance for the different codes, which can be detrimental to the prediction performance^44,45^, and secondly, different codes have very different prevalence rates which likely hurts the performance for rarer diseases disproportionately.

We adopt a conceptually simple binary relevance based classifier testing a logistic regression and a random forest pipeline base. Besides being a natural choice for modeling multi-label outputs, the binary relevance based approach provides several additional benefits. First, it allows us to account for the fact that different labels have very different prevalence. To combat class imbalance, we use majority under-sampling, which also reduces the computational expense. Also, we can calibrate each model independently based on the prevalence of the corresponding label. Another advantage to the binary relevance based approach is that the model can be easily extended to accommodate additional labels of interest—if new target labels are introduced (say, due to availability of new data over time), we only need to train one additional model for each new label. Also, if the prevalence of an individual label changes with time (many diseases show a seasonal pattern, for example), we can recalibrate only the corresponding model. In essence, the binary relevance based approach allows us to update SCOPE simply and efficiently.

Another key consideration in developing SCOPE was interpretability, particularly as it relates to transparency^12^. Logistic regression and random forests are well understood theoretically and are obviously more algorithmically transparent to deep learning models such as RNNs. We have also striven to preserve decomposability, which relates to the idea that each component of the model including the inputs, the parameters and the computational steps should be intelligible. To that end, we adopted the early stage feature integration strategy of concatenating features obtained from different modalities, avoided extensive feature preprocessing or feature engineering and chose not to use deep learning based feature embeddings. These choices ensured that each input to SCOPE is interpretable to the user. Finally, the binary relevance based approach greatly enhances post hoc interpretability, which relates to the explanations or contextual information such as feature importance that provide insight about the trained model. Since we have a separate model for each label, performing post hoc interpretation of each model separately allows us to infer how the features interact in predicting the individual label. While the notion and importance of interpretability of models has been subject to some debate^46,47^, we believe transparency can facilitate adoption of machine learning models in the clinic. It may also reduce fears of brittleness, particularly prevalent in deep learning models, where small perturbations of input may lead to wildly different predictions in edge cases^48^.

Interpretable models are particularly attractive if they do not suffer from performance penalties. Fortunately, this seems to be the case with SCOPE which performs at par or better based on our results with existing models. In particular, we note that in terms of recall$@k$, our results are at par with the best results in Choi et al.^15^ (Table 3). Also, in terms of AUROC on individual labels, our model achieves better results than those reported in Rashidian et al.^11^, and the deep learning model proposed in Choi et al.^15^ when trained on diagnostic codes only.

Our work also suggest several avenues for future research. First, SCOPE was trained, validated and tested on a single dataset obtained from Stanford hospital and clinics. While model interpretation shows that the important features are meaningful, we have not assessed the performance of this model on external datasets, nor have we tried to validate or draw causal connections between the identified important features and the predicted diagnoses from a clinical point of view. Unfortunately, to the best of our knowledge, there are no publicly available EHR datasets that track office visits (rather than hospitalization/ICU visits as MIMIC^49^ does). Despite starting with more than $2.5$ million patients, the model was developed for 583 diagnostic codes which covers only around 40% of all relevant three-character ICD-10 codes. This points to the fact that many codes are rare and more data is likely required for developing models to cover them. We dropped infrequent codes in our modeling approach. We note however, that this does not detract from the usefulness of our model since these codes are quite rare and occurred in less than 500 (out of > 500,000) patients, or less than 0.1% of our cohort. Finally, EHR data can be incomplete and biased^4^ and models developed on them can be susceptible to that bias.

In conclusion, we found that SCOPE, an RF model using diagnostic codes and labs results, can predict future outpatient visit diagnosis as well as a or better than popular deep learning benchmarks, but with greater interpretability of contributing features. The promise of SCOPE is that by predicting the likely diagnoses for a patient based on past medical history, it can guide the physicians in their pre-visit preparation or aid them fill out the post-visit documentation, and in the process potentially reduce the incidence of missing or incomplete entries in the EHRs.

## Supplementary Information

Supplementary Information.

## Data Availability

The EHR data obtained from Stanford Healthcare cannot be made publicly due to HIPAA issues and data requests should be directed to the corresponding author.
