# Supplementary material for: SCOPE: predicting future diagnoses in office visits using electronic health records
Source: Sci Rep. 2023 Jul 7;13:11005. doi: 10.1038/s41598-023-38257-9 (PMC10328934; doi:10.1038/s41598-023-38257-9)
Supplement: Supplementary file 1 — Supplementary Information. [file 41598_2023_38257_MOESM1_ESM.pdf]

Supplementary Information

Details of Materials and Methods

Data

For this study, the EHR data of 2,701,522 patients at Stanford Healthcare with 55,068,909 encounters over a time period from January 2008 to December 2016 was retrospectively collected and deidentified in accordance with approved IRB guidelines. We selected all encounters that were completed office visits. We removed infrequent ICD-10 codes that were present in fewer than 500 patients and excluded encounters for which no ICD-10 diagnosis codes were available. Furthermore, patients with only one encounter were removed. For the selected patients, we extracted demographic information (i.e. age and sex) for each patient.

Next, we extracted all the lab tests for the selected patients. We used the lab names as the identifiers and used heuristic rules such as conversion to lower case and whitespace stripping, along with manually supervised heuristics such as mapping recurring abbreviations to full names and some case-by-case determinations if two lab names referred to the same test, to harmonize them. Next, we removed infrequent lab tests that were present in fewer than 500 patients. We also harmonized the lab values. We divided the labs into two categories: numeric labs which had at least one numeric value (e.g. blood glucose and cholesterol), and non-numeric labs whose values were noted by phrases such as "detected", "not detected", "present" (e.g. respiratory virus panel tests), etc. For each numeric lab, the units of measurement were harmonized by picking the most frequently used unit as the common unit and then converting the other units to the common unit. For the non-numeric labs, heuristics were used to get binary values, "True" if present and "False", otherwise. We dropped instances for which the harmonization was not possible.

Data Modalities

In order to predict the probable diagnoses at an encounter, we considered patient demographics, past diagnoses codes, and lab results. The demographic information comprised age (in years) and sex (binary, indicating male or female) of the patient.

For both diagnosis codes and lab results, we used a simple aggregation strategy. We considered four time windows for diagnosis codes: 90, 180, 365 and 3650 days, and four time windows for lab results: 30, 90, 180 and 365 days. Given a time window, we consider the presence or absence of the diagnosis code within that time window (strictly) before the encounter as the corresponding feature. Similarly, for lab results, we consider all tests done within the predefined time window (may differ from that of the diagnosis codes). If a test was performed more than once in the window, the most recent test is considered. For numeric labs, the actual reported values were used as features, while the binary values were used for the non-numeric labs.

In order to handle missing values in the lab results, we inferred `normal values' for each lab test. For the numeric labs, the normal value was the median of all results that were deemed to fall in the normal range, as noted in the EHR. For the non-numeric labs, the mode was taken to be the 'normal value'. These normal values were used to fill missing values in encounters where the test results were unavailable. Additionally, since the very presence or absence of a lab test may be informative, we included a binary indicator variable for each lab, representing the presence or absence of the test within the time window.

Output Labels

The output labels at each encounter are the corresponding ICD-10 diagnostic codes. We do not consider codes starting with R, U, V, and Z as valid labels since they comprise either symptoms, codes for special purposes, external causes of morbidity and mortality or factors influencing health status and contact with health services. In order to avoid the large cardinality of all possible ICD-10 diagnosis codes, we grouped them under their three-character prefixes, for example, all codes starting with I25 (I25.1 – I25.9, and all codes underneath) are assigned the same label I25. One-hot encoding was used to create the output label vector for each encounter. Note that multiple labels may be present simultaneously at a single encounter. Each label is further designated as acute or chronic using the Chronic Condition Indicator for ICD-10-CM[1].

Model Architecture

We adopt a binary relevance based multi-label classification strategy using either logistic regression (LR) or random forest (RF) as the base classifier. Thus, given a set of m labels, the trained model contains m classifiers, each trained on an individual label independently. The LR pipeline comprises a majority under-sampling step (1:1 positive to negative ratio), followed by a maximum absolute scaler, followed by a logistic regression classifier with an l_1 or l_2 penalty. The RF pipeline consists of the majority under-sampling step, followed by a random forest classifier. No additional preprocessing is used with the random forest classifier. Note that though random forest can support multi-label classification directly, we did not use it here since we undersampled the negative samples to match the number of positive samples and each output label has its own prevalence.

Choice of Aggregation Windows

We built LR and RF models based on both diagnostic codes and lab results individually for each of the respective aggregation windows. For a fixed model and two candidate time windows $t_1$and t_2, t_1 is considered 'better' than t_2 if the average AUROC performance with $t_1$is significantly (at p < 0.001, Wilcoxon signed-sum rank test) better than the performance with t_2. If the difference in performance is not significant, $\min(t_1, t_2)$is considered 'better'. The best time windows were chosen for both diagnostic codes and lab results with both LR and RF models and were used for multi-modal feature integration in the next step.

Multi-modal Feature Integration

To combine features from multiple modalities (i.e. diagnosis codes and lab results), we adopted an early stage integration strategy where the respective feature vectors are concatenated to form a longer vector. First, the diagnostic codes features and the lab features, aggregated with their respective best time windows, are integrated and used to build both LR and RF models. Next, the demographic features are added to the diagnostic and the lab features. Between LR and RF, the model that performs better on the combined feature set is chosen for further analysis.

Training and Validation

The same cohort of patients was used for every prediction task. We split the full set of patients into a training (60%), a validation (20%) and a testing (20%) set. The training and validation sets are used for training the models, tuning hyperparameters and model selection. The testing set is used only for the final performance evaluation.

Choice of Evaluation Metrics

To evaluate and compare the efficacy of different models on various labels, we primarily use the Area under the Receiver Operating Characteristics Curve (AUROC) since it is insensitive to varying class imbalance across different labels. For the final model, in addition to AUROC, we employ other metrics including the Area under the Precision Recall Curve (AUPRC), and multi-label classification metrics such as recall@k and coverage error which are defined as:

$$recall@k = Average\left(\frac{Number\ of\ true\ positives\ in\ top\ k\ predictions}{Number\ of\ positive\ ground\ truth\ labels}\right)$$

$$coverage\ error = Average\left(\max_{l \in positive\ labels} rank(l)\right)$$

where $rank(l)$ refers to the rank of a label l in the sorted (in descending order) predictions. Additionally, we evaluate the performance of the final model for *de novo* predictions: for a given label and each patient, we only consider encounters till the first occurrence of the label, and evaluate the performance of our model in terms of AUROC and AUPRC.

Model Interpretation

We used the SHAP (SHapley Additive exPlanations) framework introduced in Lundberg et al.[2] for model interpretation. Shapley values allow us to examine the importance of features for a single sample, as well as overall importance by considering the average of their absolute values across all samples. To determine the Shapley values

for the RF model, we used the TreeExplainer[3] which is a computationally efficient algorithm with theoretical guarantees on consistency.

To identify important features at a chapter level, we first computed the importance of features for each label in the chapter. Then, we considered the top k=50 most important features for each label and counted how many times each of them recurred across all the labels in the chapter. Finally, the top k=50 most frequent among them were chosen to represent the important features for the chapter, and were visualized using word clouds.

Model Calibration

A calibrated model was developed using isotonic regression[4] with five-fold cross-validation on the training set. For each target label and each fold, the chosen model pipeline was fit on the training fold and then calibrated on the validation fold. Thus, we got an ensemble of five fitted calibrated models for each label with five-fold cross-validation. During prediction, the outputs of the five models were averaged. This method also has the benefit of ensembling, possibly providing more accurate and stable predictions.

References

1. *Chronic Condition Indicator (CCI) for ICD-10-CM (Beta Version)*. Rockville, MD.: Agency for Healthcare Research and Quality; 2018. https://www.hcup-us.ahrq.gov/toolssoftware/chronic_icd10/chronic_icd10.jsp. Accessed July 15, 2020.

2. Lundberg SM, Allen PG, Lee S-I. A Unified Approach to Interpreting Model Predictions. In: *Advances in Neural Information Processing Systems 30*. ; 2017:4765-4774. https://github.com/slundberg/shap. Accessed June 25, 2020.

3. Lundberg SM, Erion G, Chen H, et al. From local explanations to global understanding with explainable AI for trees. *Nat Mach Intell*. 2020;2(1):56-67. doi:10.1038/s42256-019-0138-9

4. Chakravarti N. Isotonic Median Regression: A Linear Programming Approach. *Math Oper Res*. 1989;14:303-308. doi:10.2307/3689709

Supplementary Tables

Letter prefix	Disease category
A	Certain infectious and parasitic diseases
B	Certain infectious and parasitic diseases
C	Neoplasms
D	Neoplasms and Diseases of the blood and blood-forming organs and certain disorders involving the immune mechanism
E	Endocrine, nutritional and metabolic diseases
F	Mental and behavioural disorders
G	Diseases of the nervous system
H	Diseases of the eye, ear, mastoid process, and adnexa
I	Diseases of the circulatory system
J	Diseases of the respiratory system
K	Diseases of the digestive system
L	Diseases of the skin and subcutaneous tissue
M	Diseases of the musculoskeletal system and connective tissue
N	Diseases of the genitourinary system
O	Pregnancy, childbirth and the puerperium
P	Certain conditions originating in the perinatal period
Q	Congenital malformations, deformations and chromosomal abnormalities
S	Injury, poisoning and certain other consequences of external causes
T	Injury, poisoning and certain other consequences of external causes

Supp. Table 1: Letter prefixes and corresponding disease categories

Supplementary Figures

Supp. Fig. 1: Effect of aggregation windows on AUROC performance for LR and RF models. (a) Diagnostic codes only with aggregation windows (90, 180, 365 and 3650 days) (b) Lab results only with aggregation windows (30, 90, 180 and 365 days).

Supp. Fig. 2: Performance of LR and RF with different inputs: diagnostic codes only (Diag), lab results only, combination of labs and diagnostic codes (LabsDiag) and the combination of lab results, diagnostic codes, and demographic features (LabsDiagDemo)

Supp. Fig. 3: Calibration plots for (a) Validation set (b) Test set. An RF pipeline was trained and calibrated for each label with isotonic regression

Supp. Fig. 4: Word cloud visualization of feature importances for letter prefixes (a) C (neoplasms), (b) I (Diseases of the circulatory system), (c) F (Mental and behavioural disorders), and (d) J (Diseases of the respiratory system)

Supp. Fig. 5: Summary plot visualization of feature importances for individual labels within letter prefixes C, F, I and J: (a) C34 (lung cancer), (b) I50 (heart failure), (c) F31 (bipolar disorder) and (d) J44 (chronic obstructive pulmonary disease). Higher absolute SHAP values (on average) indicates higher importance. The color red indicates high feature values. A concentration of blue points on the left of the vertical line (SHAP value impact 0) indicates that lower feature values tends to promote a negative prediction for the disease, while its presence on the right indicates that lower values tend to promote a positive prediction for the disease.

Supp. Fig. 6: Chapterwise AUROC Performance of DL vs LR vs RF on de novo predictions. The p-values are obtained by a paired Wilcoxon signed-rank test with the alternative hypothesis that DL is better on overage.

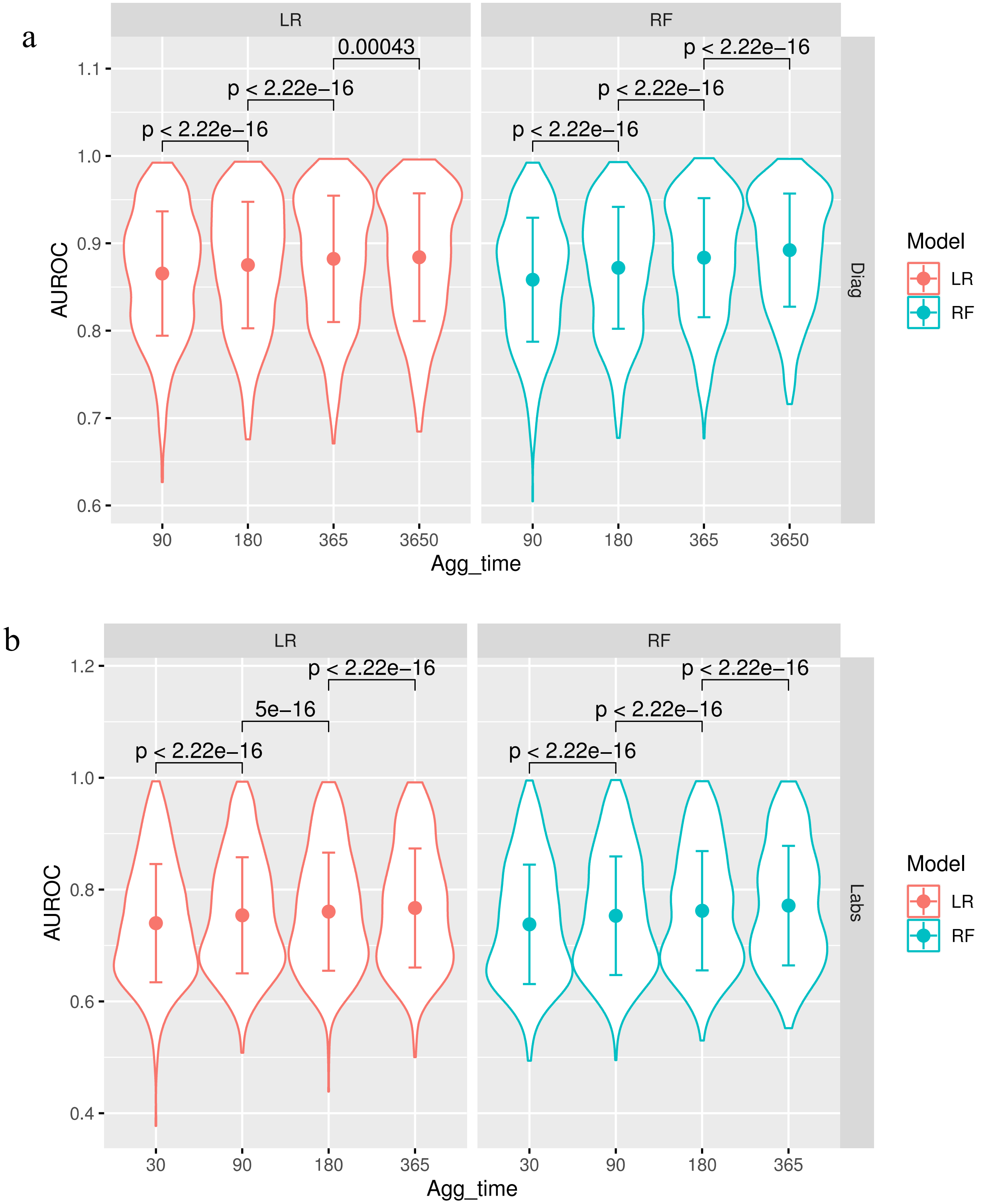

Supp. Fig. 1: Effect of aggregation windows on AUROC performance for LR and RF models. (a) Diagnostic codes only with aggregation windows (90, 180, 365 and 3650 days) (b) Lab results only with aggregation windows (30, 90, 180 and 365 days).

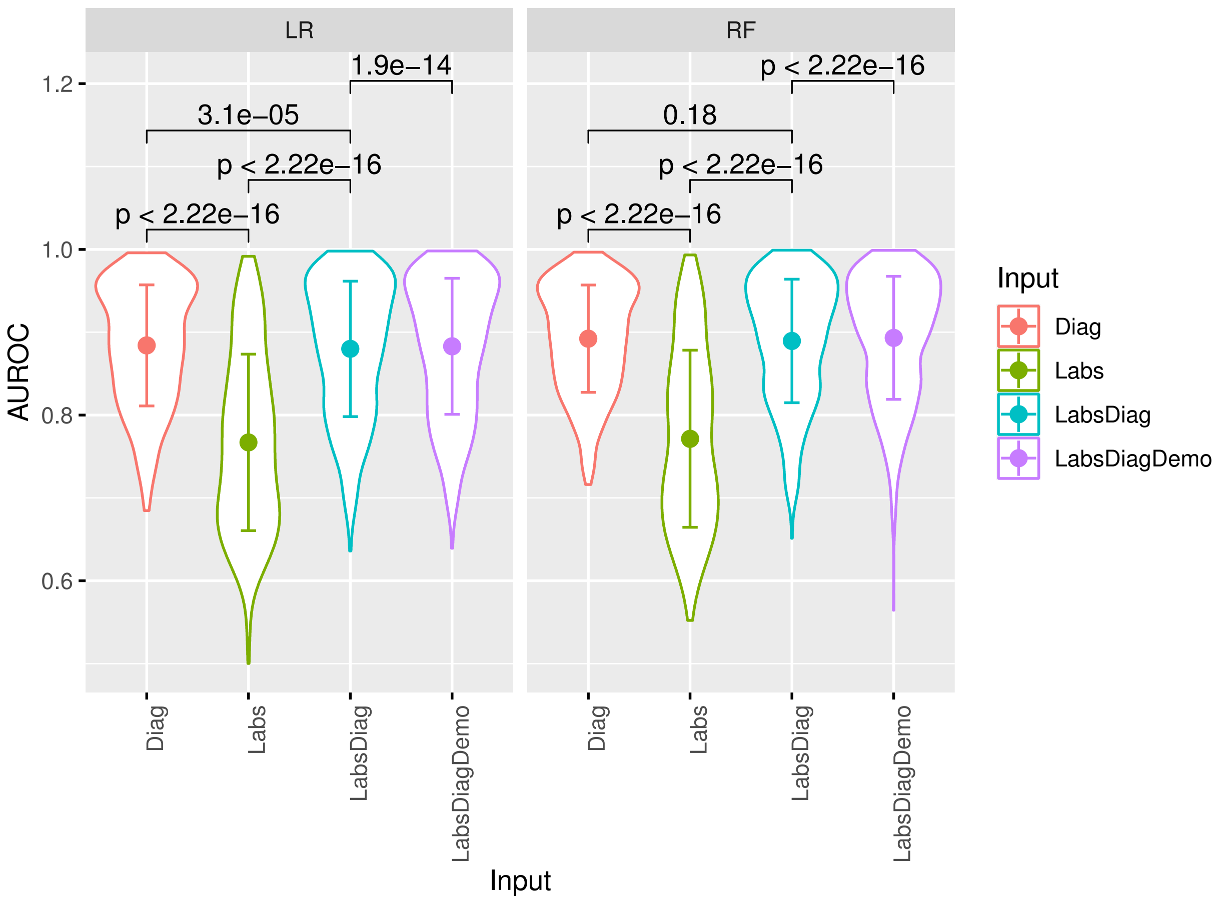

Supp. Fig. 2: Performance of LR and RF with different inputs: diagnostic codes only (Diag), lab results only, combination of labs and diagnostic codes (LabsDiag) and the combination of lab results, diagnostic codes, and demographic features (LabsDiagDemo)

a

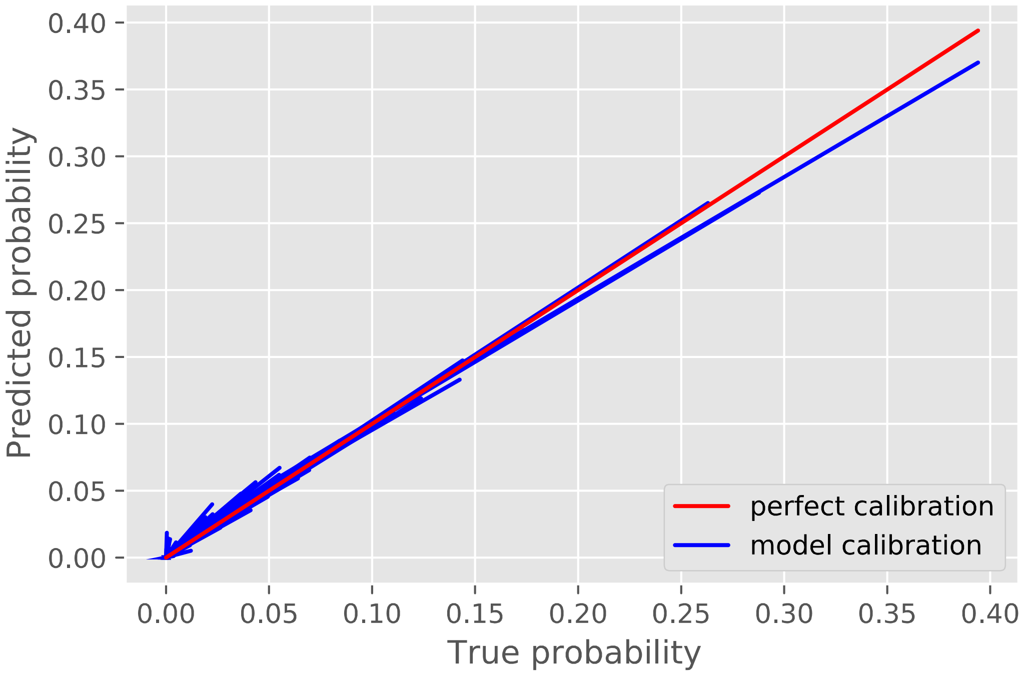

b

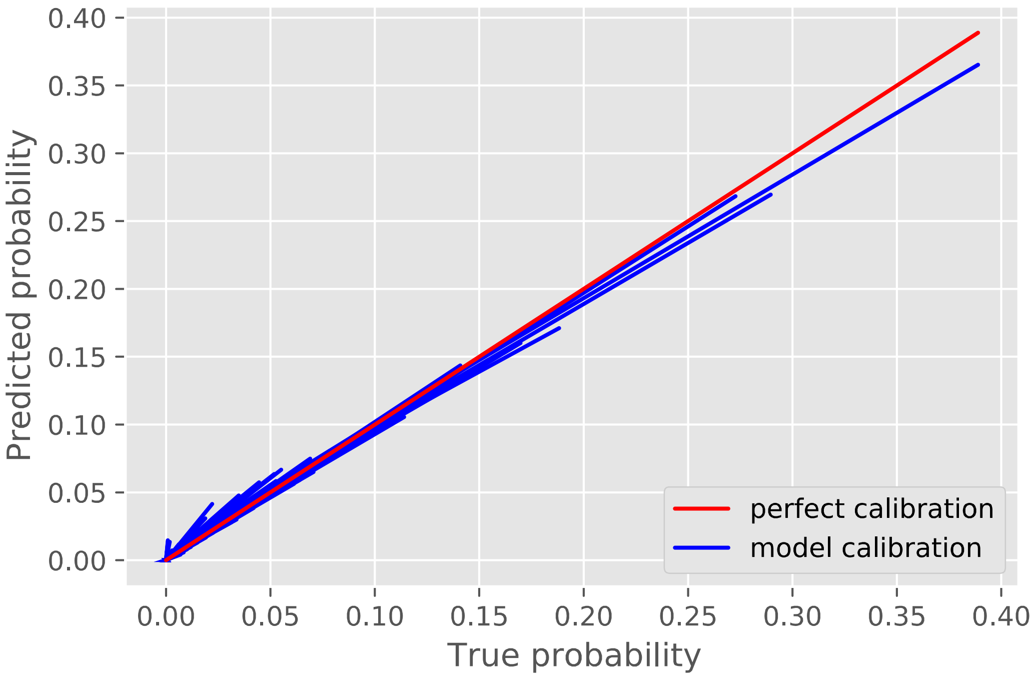

Supp. Fig. 3: Calibration plots for (a) Validation set (b) Test set. An RF pipeline was trained and calibrated for each label with isotonic regression

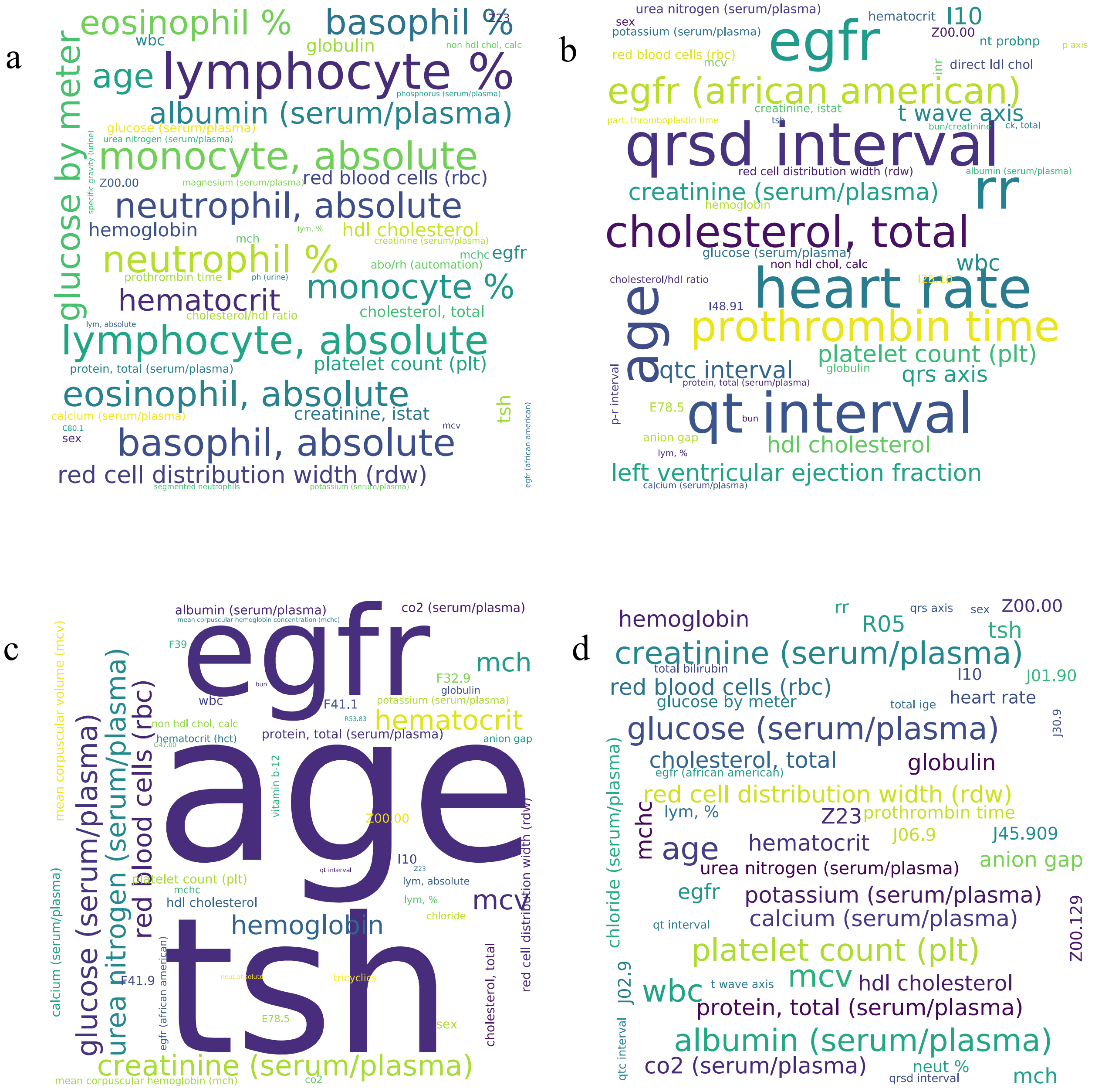

Supp. Fig. 4: Word cloud visualization of feature importances for letter prefixes (a) C (neoplasms), (b) I (Diseases of the circulatory system), (c) F (Mental and behavioural disorders), and (d) J (Diseases of the respiratory system)

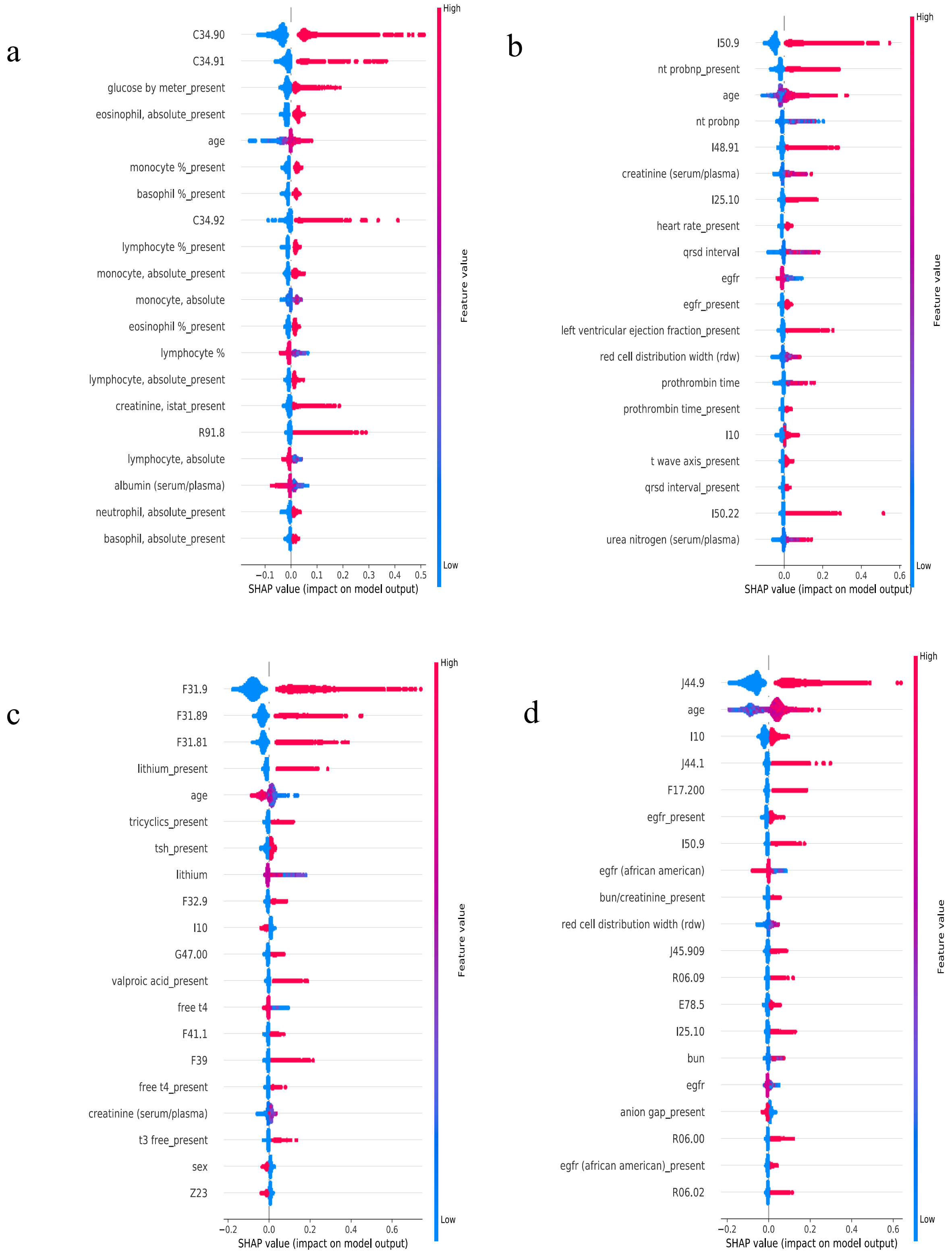

Supp. Fig. 5: Summary plot visualization of feature importances for individual labels within letter prefixes C, F, I and J: (a) C34 (lung cancer), (b) I50 (heart failure), (c) F31 (bipolar disorder) and (d) J44 (chronic obstructive pulmonary disease).

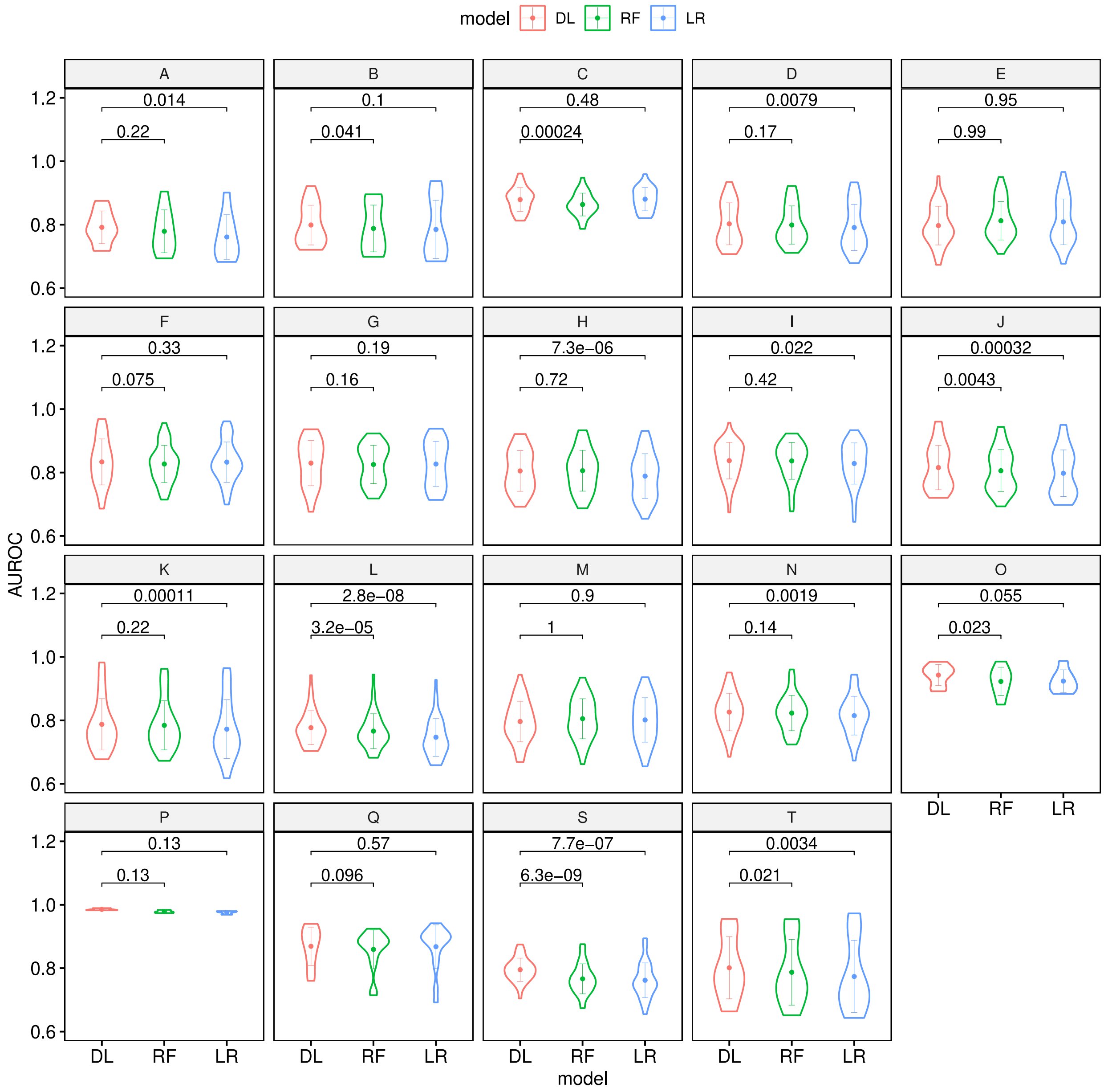

Supp. Fig. 6: Chapterwise AUROC Performance of DL vs LR vs RF on de novo predictions. The p-values (numbers on the top brackets) are obtained by a paired Wilcoxon signed-rank test with the alternative hypothesis that DL is better on overage.
